# MicroRNA‐125b as a tumor suppressor by targeting MMP11 in breast cancer

**DOI:** 10.1111/1759-7714.13441

**Published:** 2020-04-14

**Authors:** Yanan Wang, Yaning Wei, Xiangyu Fan, Pei Zhang, Pan Wang, Shujie Cheng, Jinku Zhang

**Affiliations:** ^1^ Department of Pathology Affiliated Hospital of Hebei University Baoding China; ^2^ Department of Oncology Affiliated Hospital of Hebei University Baoding China; ^3^ Department of Surgery Affiliated Hospital of Hebei University Baoding China; ^4^ Department of Pathology No.1 Central Hospital of Baoding Baoding China

**Keywords:** Breast cancer, MicroRNA‐125b, migration, MMP11

## Abstract

**Background:**

Breast cancer is a common type of tumor in women worldwide. MicroRNAs have been identified as regulators in many human cancers. The aim of this study was therefore to investigate the functional role of miR‐125b in regulating breast cancer progression.

**Methods:**

We used the StarBase database to investigate the expression of miRNA‐125b in breast cancer and adjacent normal tissues. MMP11 3′‐UTR construct and luciferase reporter assays was performed for target genes. Cell proliferation was evaluated by CCK‐8 and colony formation assay. The migration and invasion were assessed by transwell assay.

**Results:**

Luciferase reporter assays showed miRNA‐125b directly targeted MMP11. miRNA‐125b by transfection with its mimic in breast cancer cells significantly suppressed breast cancer cell proliferation and migration. Western blot revealed that overexpression of miRNA‐125b substantially reduced MMP11 protein expression. We used the UALCAN database to investigate the expression of MMP11 in human breast cancer and adjacent normal tissues. In addition, we found that miRNA‐125b spoiled MMP11 induced breast cancer cell proliferation and migration promotion effect.

**Conclusions:**

miRNA‐125b mimic inhibited proliferation, migration, and invasion of breast cancer cells through targeting MMP11 protein.

## Introduction

Breast cancer is the most common malignant tumor in women worldwide.^1^ According to statistics data from the World Health Organization, 12 million new breast cancer patients are added each year, causing 465 000 deaths, and the prevalence of breast cancer continues to rise.^2^, ^3^

Matrix metalloproteinases (MMP) are a family of Zn^2+^‐ dependent endonucleases involved in the degradation of extracellular matrix. At present, there are 28 protein members in the MMP family. They can be divided into the following subgroups which include collagenase and gelatinase, interstitial lysins, and membrane‐type MMPs.^4^, ^5^ MMP‐11 was first discovered by Basset *et al*. in 1990 and MMP‐11 was considered to play an important role in the development of breast cancer.^6^ MMP‐11 is different from other proteases of the MMP family in that it is secreted as an active precursor and can only degrade some enzymes (such as serine proteases), and cannot directly hydrolyze extracellular matrix molecules. Some studies have found that MMP‐11 may be involved in the invasion and metastasis of breast cancer, but the functional role of MMP‐11 is still unclear. In our study, we investigated the roles of MMP11 in breast cancer cells.

MicroRNA is a small molecule RNA formed by Dicer enzyme processing in eukaryotes, and has high conservation, timing and tissue specificity.^7^ MicroRNA is ubiquitous in different organisms, and is closely related to the occurrence and development of tumors. Mainstream research suggests that mature microRNAs, together with other proteins, form an RNA‐induced silencing complex that binds to the 3′untranslated region (3′UTR) of the target gene mRNA in a fully or partially complementary manner and silences gene expression. Studies have found that at least one third of the protein‐coding genes are regulated by microRNAs, and their regulation almost involve the entire process of cell life, including differentiation, proliferation, metabolism and apoptosis.^8^, ^9^ Therefore, a microRNA has many downstream target genes that can cause extensive protein changes, thereby inducing or suppressing the occurrence or development of tumor. A large number of studies have shown that miRNA‐125b is significantly increased in tumor tissues of oral and endometrial cancers, and can promote tumor cell proliferation through various pathways.^10^, ^11^ MicroRNA, as a population with significant abnormal expression, regulates mRNA expression mainly at the post‐transcriptional level. Numerous tumor‐related proteins are regulated by microRNAs in this process, and play a role in regulating tumor nutrition and vascular supply, inducing immune escape, and changing the microenvironment of tumors. As an early confirmed abnormal expression molecule in invasive breast cancer, the relationship between miR‐125b and some proteins that are clearly involved in tumor cell proliferation and metastasis has been a hot topic in recent years. It has been reported that inhibiting miR‐125b regulates the Wnt/β‐catenin pathway and EMT.^12^ In many breast cancer cell lines, miR‐125b inhibited BAK1 gene expression and confers paclitaxel resistance.^13^ In addition, it has been confirmed that miR‐125b can induce distant metastasis of breast cancer by mediating the STARD13 in MCF‐7 and MDA‐MB‐231 breast cancer cell lines.^14^ However, the relationship between miR‐125b expression and breast cancer has not as yet been fully clarified, and more detailed mechanism research is needed to clarify its complex biological effects. In this study, we demonstrated that miR‐125b can regulate MMP11 expression to fully illustrate the mechanisms involving breast cancer progression.

## Methods

### Cells and reagents

The human breast cancer cell lines T47D and SKBR3 were purchased from the Shanghai Cell Bank of the Chinese Academy of Science (Shanghai, China). All breast cancer cell lines were incubated in DMEM (Invitrogen, Thermo Fisher Scientific, MA, USA). All cells were cultured in an incubator containing 5% CO_2_, and 10% fetal bovine serum was added to the cell medium. The cell culture temperature was 37°C. The synthetic miRNA fragments (Rebio, Shenzhen, China) were transfected using Lipofectamine 3000 (Invitrogen, Thermo Fisher Scientific). The MMP11 recombinant plasmid and negative control plasmid (Origene, Suzhou, China) were added to the cell lines according to the manufacturer's protocol. For the following experiments, the cells were harvested after 48 hours of transfection. The antibodies used for immunoblotting were as follows: GAPDH (ab8245), MMP11 (ab53143) (Abcam, Cambridge, USA), ACTB (3700) (Cell Signaling Technology, Danvers, USA).

### Western blot

Cells were digested with 0.25% trypsin and collected by centrifugation (1000 *g*), then lysed in RIPA buffer for 30 minutes. Protein concentration was determined using a bicinchoninic acid protein determination kit (Thermo Scientific, MA, USA), and then mixed with 1x loading buffer. The sample proteins were separated using 10% sodium dodecyl sulfate‐polyacrylamide gel electrophoresis (SDS‐PAGE) and the proteins were blotted onto polyvinylidene fluoride (PVDF) membranes (Millipore, Billerica, MA, USA). The membranes were blocked with 5% nonfat skimmed milk for one hour at room temperature, incubated overnight at 4°C with primary antibody, and then incubated with secondary HRP conjugated antibody for one hour at room temperature. The immunoreactive blots were visualized using an enhanced chemiluminescence reagent (Beyotime, Shanghai, China).

### Quantitative real‐time PCR (qRT‐PCR)

Total RNA was extracted from breast cancer cell lines using TRIzol Reagent (Life Technologies, Carlsbad, CA, USA). The cell growth medium was removed and 1 mL of TRIzol Reagent per 10^5^ cells was added to the culture dish to lyse the cells. It was then incubated for five minutes to permit complete dissociation of the nucleoproteins complex. Then, 200 μL of chloroform per 1 mL of TRIzol was added and incubated for two minutes. The sample was centrifuged for 15 minutes at 12 000×*g* at 4°C. The aqueous phase containing the RNA was then collected and we proceeded to precipitate, wash and then solubilize the RNA. For reverse transcription (RT) of microRNA, we used miScript reverse transcription kit (Qiagen, Dusseldorf, Germany). This was then quantified with SYBR‐green real‐time Master Mix under an Applied Biosystems 7900 Sequence Detection system (Applied Biosystems, MA, USA). The relative expression of miR‐125b was normalized to that of GAPDH using the 2 − ΔΔCt method.

### Cell counting kit‐8 (CCK‐8) assay

The cells were seeded in a 96‐well plate at a density of 2000 cells/well in 100 μL of culture medium. The plate was then placed for an appropriate length of time in the incubator. We then added 10 μL of CCK‐8 solution to each well of the plate. The optical density value (OD) at 450 nm was detected using a microplate spectrophotometer (Thermo Scientific, MA, USA).

### Colony formation assay

For the colony formation assay, a total of 500 cells/well were seeded into 6‐well plates and incubated for 15 days. Cell colonies were fixed with methanol, then the colonies were stained with 0.5% crystal violet (Sigma, Darmstadt, Germany) for 30 minutes at room temperature. The total number of colonies were photographed and counted.

### Transwell assay

For invasion assay, Matrigel‐coated transwell chambers (8 μm in pore size, 24 wells) were used. Cells were plated into the upper chambers with FBS‐free medium. The lower chambers were filled with medium plus with 10% FBS. After 48 hours incubation, cells on the upper surface of the insert (noninvasive cells) were removed with a cotton swab. Cells invaded by Matrigel were stained with 0.1% crystal violet and counted under a microscope.

### Wound healing assay

Transfected cells were seeded into 6‐well plates at a density of 1 × 10^5^ cells/well in medium containing 10% FBS and cultured until ~80% confluence. Six hours later, with cell confluency reaching about 90%, a 10 μL pipette was used to scratch across the surface to make a wound. The cells were then washed twice with PBS to remove cell debris. With refreshed medium, cells were incubated for 24 or 48 hours. Wounds were analyzed using Image J software.

### Luciferase reporter assay

Bioinformatic analysis algorithm TargetScan was used to predict the targets of miR‐125b. The wild‐type (wt) or mutant (mut) miR‐125b binding sequences from MMP11 3′UTR were cloned into pGL3 Basic vector. miR‐125b sequences were cloned into mCherry vectors. Thereafter, the cells were cotransfected with miR‐125b mimic, MMP11 (wt) and MMP11 (mut) using Lipo3000 according to the manufacturer's protocol. Renilla luciferase was used as a control for transfection efficiency. Cells were harvested after 48 hours incubation. The luciferase activities were analyzed using the dual‐luciferase reporter assay system (Promega, Madison, USA) after transfection for 48 hours. In brief, we prepared a sufficient amount of the 1X working concentration by adding one volume of 5X passive lysis buffer to four volumes of distilled water and mixing well. The culture plates were gently shaken and placed on an orbital shaker to ensure coverage of the cell monolayer with 1X PLB. The culture plates were then shaken for 15 minutes at room temperature. We prepared the Luciferase Assay Reagent II by resuspending the provided lyophilized Luciferase Assay Substrate in 10 mL of the supplied Luciferase Assay Buffer II. An adequate volume was prepared in order to perform the desired number of DLR assays. Then, we added one volume of 50X Stop & Glo Substrate to 50 volumes of Stop & Glo Buffer in a glass. We carefully transferred up to 20 μL of cell lysate into the luminometer tube containing LARII and mixed by pipetting, then placed the tube in the luminometer and initiated reading. We then added 100 μL Stop & Glo Reagent, mixed the solutions by vortexing briefly and initiated reading.

### Statistical analysis

If differences were found, the Student's *t*‐test was used for comparison between the two groups. Statistically significant differences among the three or above groups were determined using one‐way analysis of variance and Tukey's post hoc test. Data analysis was performed with GraphPad Prism 5 software (Graphpad Software, Inc., La Jolla, CA) and presented as means ± standard deviation (SD). If the *P*‐value did not exceed 0.05, the data was considered to be statistically significant.

## Results

### Overexpression of miR‐125b abrogates breast cancer cell proliferation

**Figure 1:**
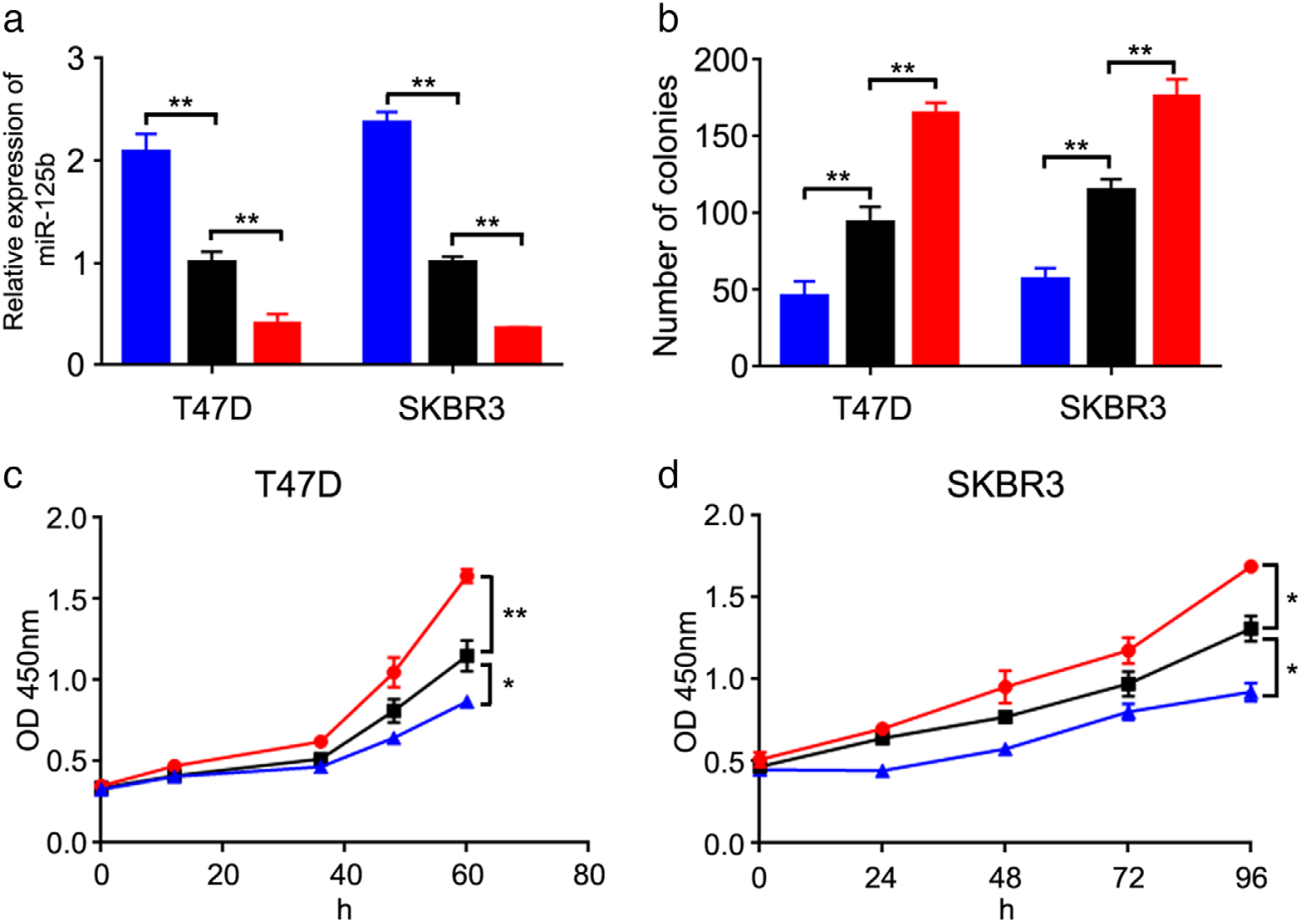
Overexpression of miR‐125b abrogates breast cancer cell proliferation. (**a**) miR‐125b expression in breast cancer cell lines (T47D and SKBR3) was measured by qRT‐PCR (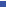) miR‐125b mimic, (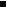) NC‐miRNA, and (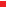) miR‐125b inhibitor. (**b**) Colony formation numbers in T47D and SKBR3 cell lines was investigated after transfecting miR‐125b mimic and inhibitor (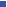) miR‐125b mimic, (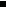) NC‐miRNA, and (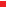) miR‐125b inhibitor. (**c**) Cell proliferation was detected in T47D after transfecting miR‐125b mimic and inhibitor (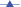) miR‐125b mimic, (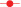) miR‐125b inhibitor, and, (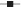) NC‐miRNA. (**d**) Cell proliferation was detected in SKBR3 after transfecting miR‐125b mimic and inhibitor (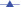) miR‐125b mimic, (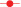) miR‐125b inhibitor, and, (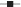) NC‐miRNA. **P < 0.01, ***P < 0.001.

The synthetic miRNAs were transfected into the T47D and SKBR3 cell lines. The results of qRT‐PCR showed that expression level of miR‐125b was obviously increased by miR‐125b mimic but decreased by miR‐125b inhibitor (Fig 1a). The colony formation assay exhibited that miR‐125b mimic reduced the number of colonies and miR‐125b inhibitor suppressed the colony formation ability of breast cancer cells (Fig 1b). In addition, the CCK‐8 assay exhibited that miR‐125b mimic inhibited, whereas miR‐125b inhibitor enhanced breast cancer cell proliferation (Fig 1c,d). These results indicated that miR‐125b had an ability to suppress cell proliferation in breast cancer.

### Overexpression of miR‐125b suppresses breast cancer cell migration

**Figure 2:**
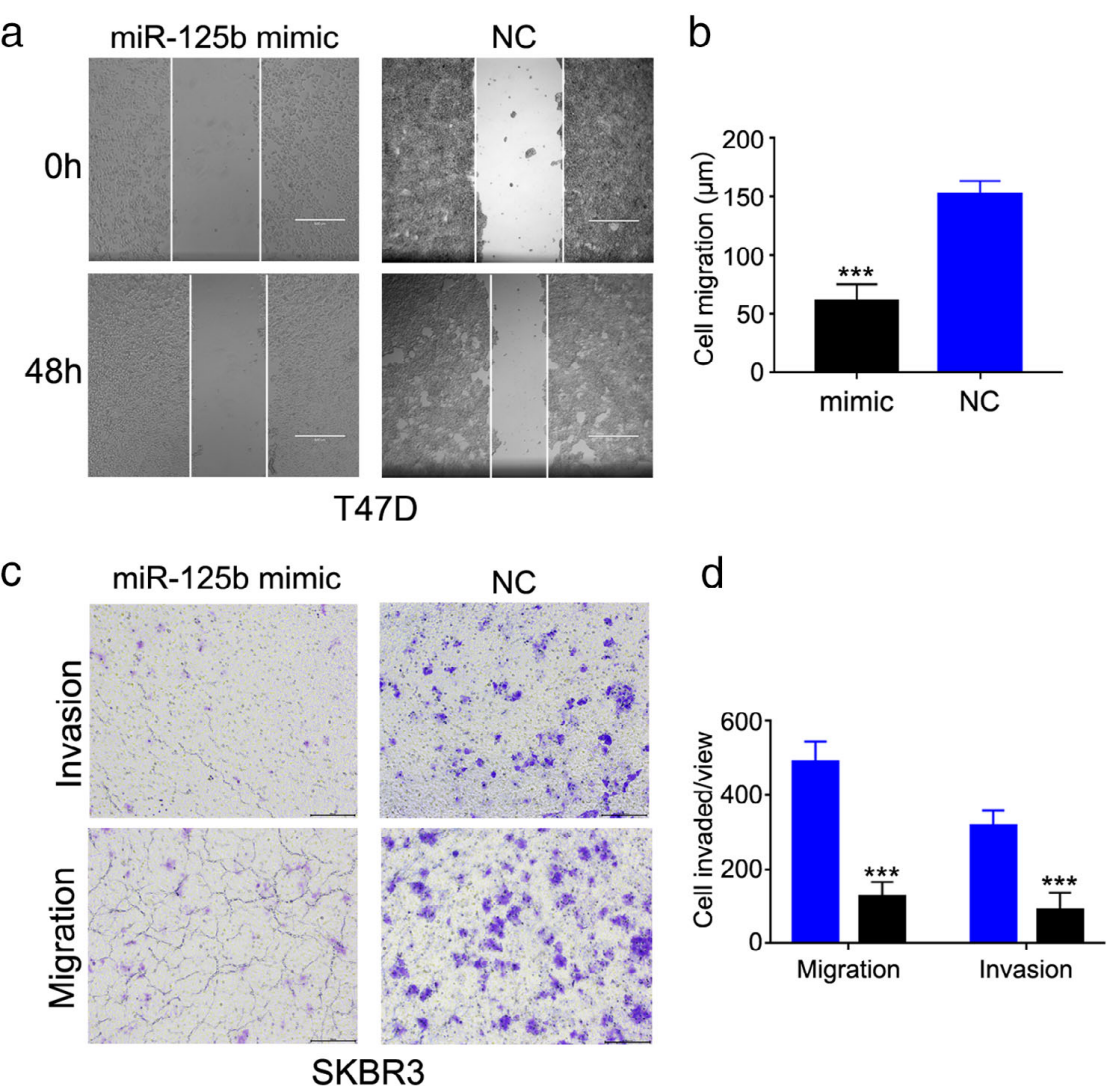
Overexpression of miR‐125b suppresses breast cancer cell migration. (**a**) Wound healing assay was performed in T47D after transfecting miR‐125b mimic. (**b**) Statistical analysis of cell migration in T47D ***P < 0.001. (**c**) Transwell assay was performed in SKBR3 after transfecting miR‐125b mimic (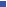) NC, and (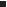) mimic. (**d**) Statistical analysis of cell migration and invasion in SKBR3, ***P < 0.001.

Next, we investigated whether miR‐125b was involved in breast cancer cell migration and invasion. Wound healing assay showed that miR‐125b mimic obviously inhibited T47D cell migration compared to control group cells (Fig 2a,b). Cell migration and invasion ability were detected by performing transwell assays following transfection with miR‐125b mimic. The data implied that miR‐125b mimic significantly reduced the migration and invasion SKBR3 cell numbers compared with the NC‐miRNA transfected group (Fig 2c,d). The results implied that miR‐125b had an ability to inhibit cell migration and invasion in breast cancer.

### miR‐125b targets MMP11 in breast cancer cells

**Figure 3:**
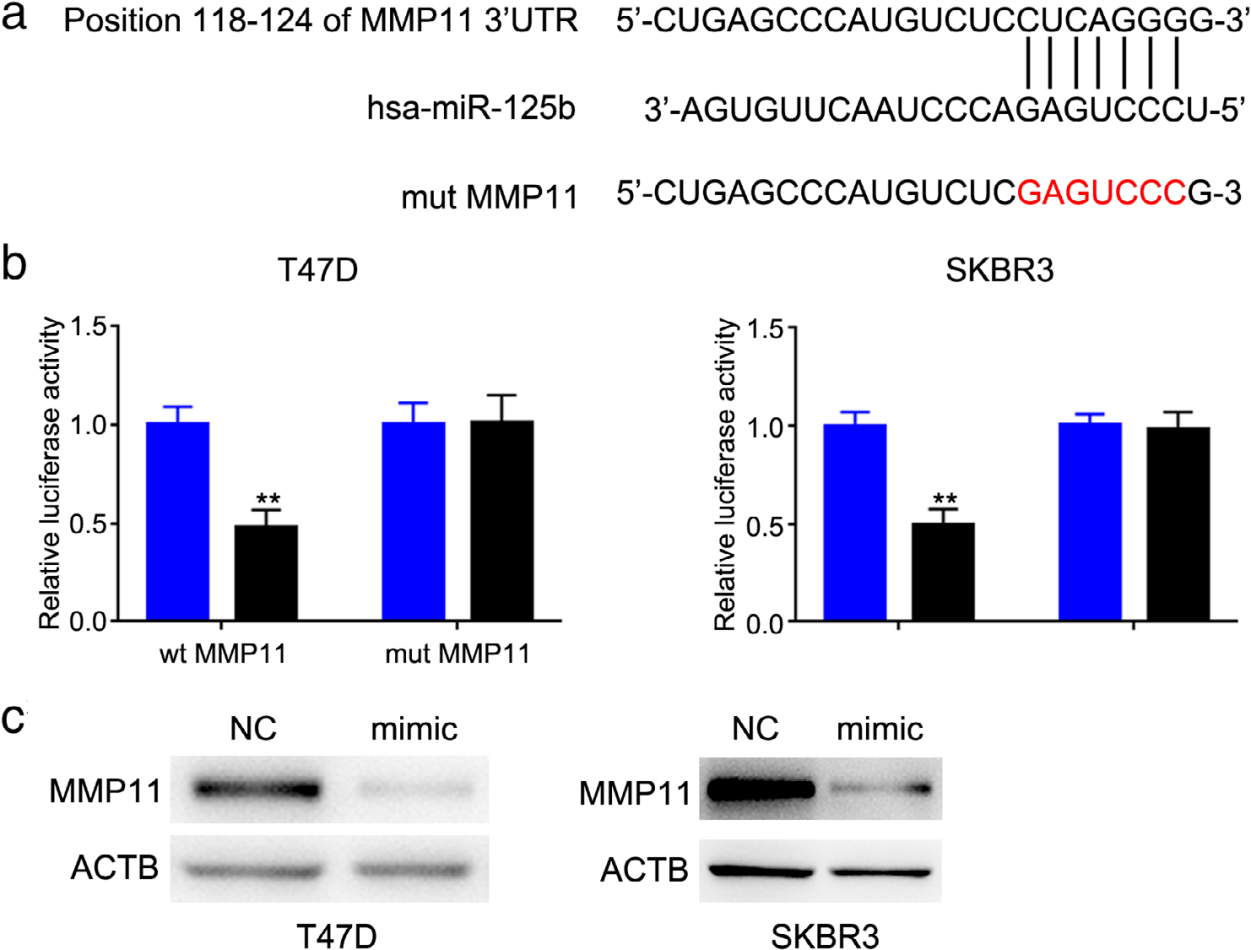
miR‐125b targets MMP11 in breast cancer cells. (**a**) Predicted binding site between miR‐125b and 3′‐UTR of MMP11. (**b**) Luciferase activity in T47D (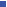) NC, and (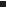) mimic and SKBR3 (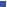) NC, and (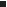) mimic cell lines transfected with wild‐type or mutation MMP11 and mimic or NC‐miRNA. (**c**) MMP11 protein expression was detected in T47D and SKBR3 cell lines transfected with miR‐125b mimic or NC‐miRNA.

To determine the putative miR‐125b target genes involved in the behavior of breast cancer cells, we combined the databases (miRBase and Targetscan). The data revealed that MMP11 contains a putative binding site for miR‐125b (Fig 3a). To confirm whether MMP11 is a direct target of miR‐125b, a renilla‐luciferase reporter assay was performed. We cloned the full length 3′UTR sequence of MMP11 mRNA containing a wild‐type or mutant miR‐125b binding sequence downstream of firefly luciferase in the dual‐luciferase vector. We detected the effect of miR‐125b on luciferase activity in 293T cells. Dual‐luciferase reporter assay showed that miR‐125b mimic could inhibit the luciferase activity of MMP11 reporters (Fig 3b). Further, we found MMP11 protein levels could be reduced by miR‐125b mimic (Fig 3c). These results showed that MMP11 was a direct target of miR‐125b in breast cancer cells.

### Restoration of MMP11 spoiled the inhibitory effects of miR‐125b on breast cancer cells

**Figure 4:**
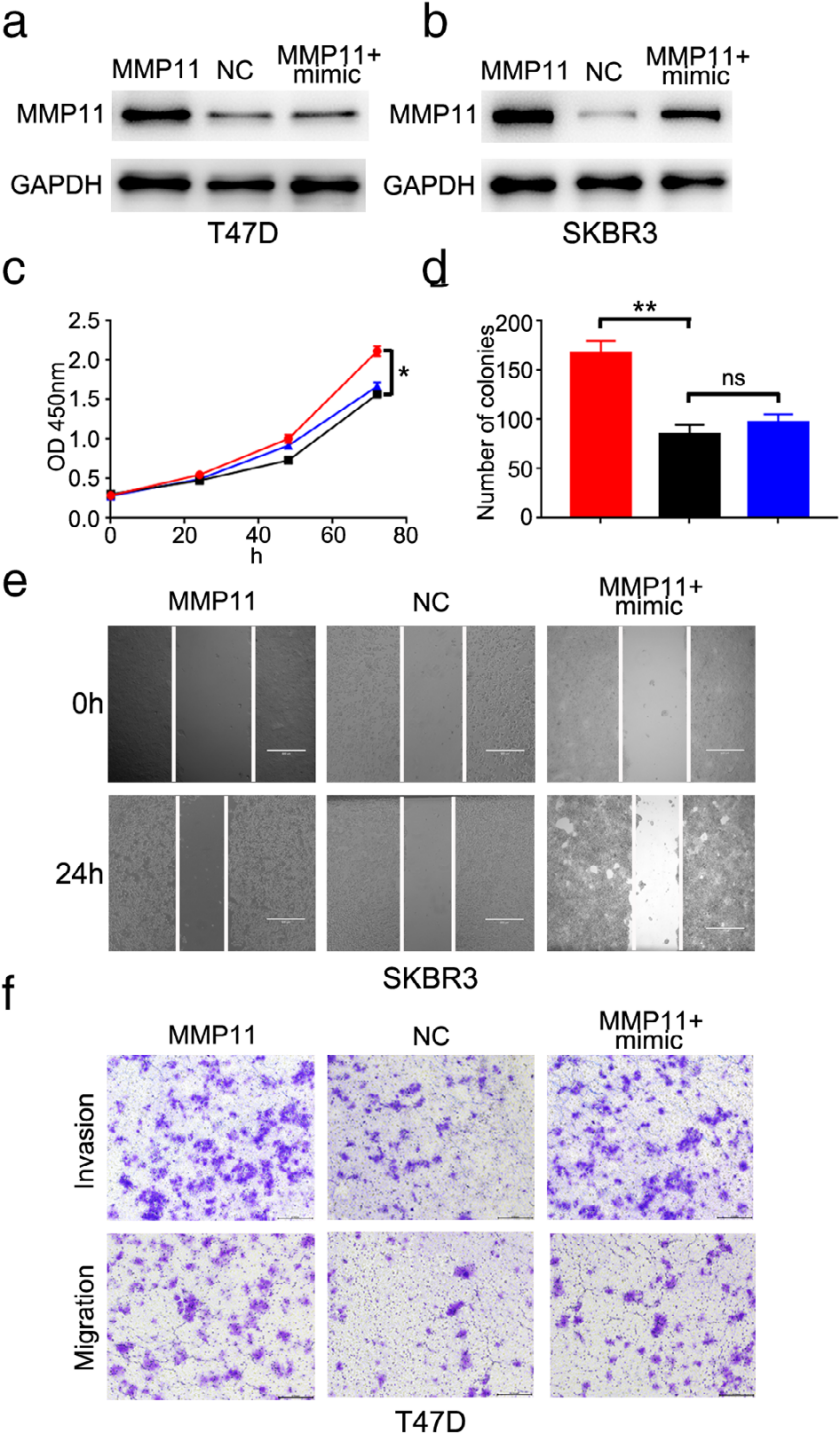
Restoration of MMP11 spoiled the inhibition effects of miR‐125b on breast cancer cells. (**a**) MMP11 protein expression in T47D cell line transfected with MMP11 construct and/or miR‐125b mimic. (**b**) MMP11 protein expression in SKBR3 cell line transfected with MMP11 construct and/or miR‐125b mimic. (**c**) Cell proliferation was detected in T47D after transfecting MMP11 construct and/or miR‐125b mimic (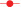) MMP11, (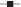) NC, and (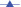) MMP11+mimic. (**d**) Colony formation numbers was detected in SKBR3 after transfecting MMP11 construct and/or miR‐125b mimic (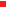) MMP11, (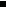) NC, and (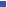) MMP11+mimic. (**e**) Wound healing assay was performed in SKBR3 after transfecting MMP11 construct and/or miR‐125b mimic. (**f**) Transwell assay was performed in T47D after transfecting MMP11 construct and/or miR‐125b mimic.

If MMP11 serves as the functional link of miR‐125b in breast cancer cells, re‐expression of MMP11 in miR‐125b overexpressed cells should be able to reverse the effects of miR‐125b. Western blot showed that MMP11 protein levels were obviously upregulated by transfecting MMP11 plasmid. Moreover, we found the upregulation effect of MMP11 plasmid could be reversed by miR‐125b mimic (Fig 4a,b). CCK‐8 and colony formation assays revealed that miR‐125b mimic abolished the promoter effects of MMP11 on cell proliferation (Fig 4c,d). The wound healing assay revealed that the facilitating effects of MMP11 on SKBR3 cell migration could be reversed by miR‐125b mimic (Fig 4e). Further, the transwell assay revealed that the promoter effects of MMP11 on T47D cell invasion and migration could also be reversed by miR‐125b mimic (Fig 4f). These findings indicate that MMP11 is a functional mediator for miR‐125b in breast cancer cells.

### Expression level of miR‐125b and MMP11 expression in breast cancer tissues

**Figure 5:**
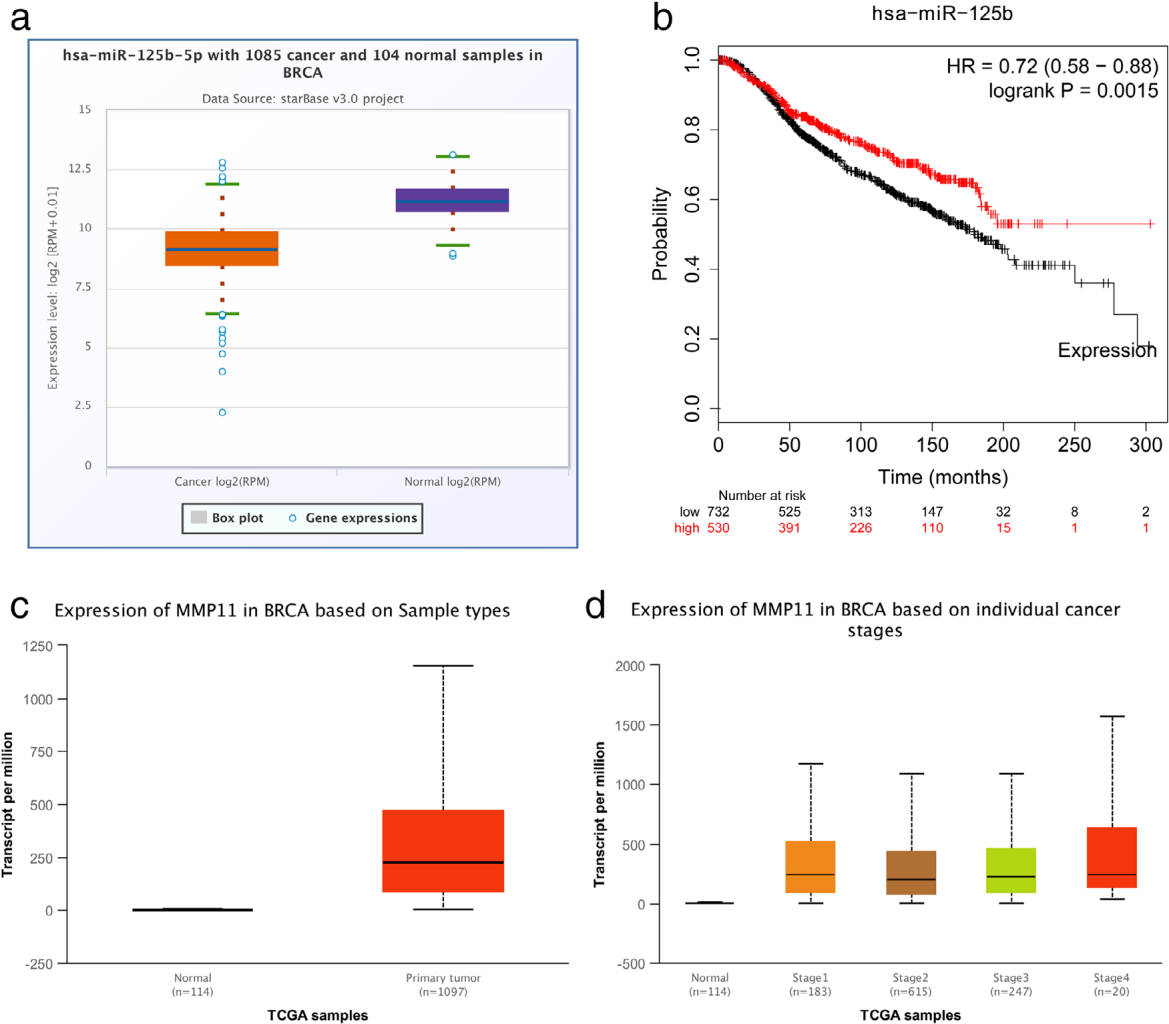
The expression level of miR‐125b and MMP11 expression in breast cancer tissues. (**a**) Low miR‐125b expression in breast cancer compared with the normal group. (**b**) Relationship between miR‐125b expression level and prognosis of patients with breast cancer (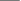) low, and (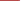) high. (**c**) High MMP11 expression in breast cancer compared with the normal group. (**d**) The expression of MMP11 was higher in breast cancer based on individual cancer stages than normal tissues.

We performed an analysis to determine miR‐125b expression in breast cancer tissues using the StarBase database. Statistical analysis of miR‐125b expression revealed that it was decreased in breast cancer tissue compared with normal breast tissue (Fig 5a). Furthermore, Kaplan‐Meier plotter database analysis showed two survival curves for the two groups defined by high and low expression of miR‐125b in patients with breast cancer, and found that miR‐125b expression level had a significant effect on overall survival of patients (Fig 5b). In addition, the expression of MMP11 between breast cancer tissue with normal breast tissue were initially analyzed by using UALCAN database (Fig 5c). Further analysis found that expression of MMP11 was significantly elevated in various individual cancer stages tissue than normal breast tissues (Fig 5d). These data were also consistent with the in vitro results.

## Discussion

The functions of some miRNAs in cancer show different characteristics: some appear as tumor suppressor genes in solid tumors, and others act as oncogenes in hematopoietic system tumors. For example, miR‐29a is involved in tumor immune escape in some solid tumors such as neuroblastoma, breast cancer, lung cancer, and prostate cancer.^15^ In addition, miR‐29a can lead to the development of acute myeloid leukemia by changing the differentiation of bone marrow progenitor cells.^16^ There are two precursors to miR‐125b: has‐miR‐125b‐1 is transcribed from chromosome 1 lq23, and its coding gene is located in an exon region; has‐miR‐125b‐2 is transcribed from chromosome 21q2, and its coding gene is located in an intron region. MiR‐125b has also very different functions in different tumors. In ovarian cancer, miR‐125b can induce cell cycle arrest and inhibit cell proliferation and clonal formation.^17^ In bladder cancer, miR‐125b intervening E2F3‐Cyclin A2 pathway regulates cell‐phase transitions, and inhibits bladder cancer cell activity and growth.^18^ miR‐125b also inhibits G1/S phase transition and cell proliferation in hepatocellular carcinoma, and has a tumor suppressor role.^19^ In contrast, colorectal cancer patients with high miR‐125b expression have poor prognosis.^20^ In nervous system tumors, miR‐125b participates in important processes in early neural differentiation.^21^ Downregulation of miR‐125b can also inhibit the proliferation of glioma cells and enhance trans retinoic acid‐induced apoptosis.^22^ In the hematopoietic system, miR‐125b may be a carcinogen. Overexpression of miR‐125b in mice can inhibit forward differentiation of bone marrow progenitor cells and cause metastatic myeloid leukemia.^23^ Transplantation of ectopically expressed miR‐125b fetal liver cells into mice can cause an abnormal increase of white blood cells and the occurrence of various blood diseases including leukemia, and indicates that miR‐125b is involved in the process of hematopoiesis.^24^ In summary, miR‐125b is a multifunctional miRNA that exerts different physiological functions through different regulatory mechanisms in different tissue systems and their tumors. The results of this study confirmed that transiently high expression miR‐125b can inhibit cell migration and proliferation in breast cancer cells, thereby demonstrating the anticancer effect of miR‐125b in breast cancer.

MMP11 was first discovered in breast cancer and has antiapoptotic functions. It can inhibit cancer cell apoptosis and promote tumor development.^25^ There are many conflicting conclusions about the correlation between MMP‐11 and clinical features, which may be related to experimental design, pathological type, ethnic type, enrolled sample size and evaluation criteria. Due to the complexity of breast cancer development and individual differences in gene expression, MMP‐11 is likely to participate in multiple steps of breast cancer development and tumor cell invasion and metastasis. In pancreatic cancer cells, MMP11 is associated with cancer cell migration and invasion, and overexpression of MMP11 can promote the occurrence and development of pancreatic cancer. This study found that MMP11 was significantly upregulated in breast cancer tissues. In breast cancer cells, MMP11 is overexpressed, with a significant increase in cell migration and invasion. Supplementing miR‐125b can reverse the effect of MMP11 on cell migration. In summary, the inhibition of MMP11 may become an intervention method for the treatment of malignant tumors.

Our data indicated that miR‐125b expression was downregulated in breast cancer tissue and mediated cell proliferation, migration and invasion through regulating MMP11, implying that the tumor suppressive role of miR‐125b in breast cancer. Targeting MMP11/miR‐125b axis may be a treatment strategy in the future.

## Disclosure

The authors declare no competing financial interests.
